# Upregulation of APP, ADAM10 and ADAM17 in the Denervated Mouse Dentate Gyrus

**DOI:** 10.1371/journal.pone.0084962

**Published:** 2014-01-03

**Authors:** Domenico Del Turco, Jessica Schlaudraff, Michael Bonin, Thomas Deller

**Affiliations:** 1 Institute of Clinical Neuroanatomy, Neuroscience Center, Goethe-University, Frankfurt/Main, Germany; 2 Department of Medical Genetics, Institute of Human Genetics, University of Tübingen, Tübingen, Germany; Nathan Kline Institute and New York University School of Medicine, United States of America

## Abstract

The disintegrin and metalloproteinases ADAM10 and ADAM17 are regarded as the most important α-secretases involved in the physiological processing of amyloid precursor protein (APP) in brain. Since it has been suggested that processing of APP by α-secretases could be involved in the reorganization of the brain following injury, we studied mRNA expression of the two α-secretases *Adam10* and *Adam17*, the ß-secretase *Bace1*, and the *App*-gene family (*App, Aplp1, Aplp2*) in the dentate gyrus of the mouse following entorhinal denervation. Using laser microdissection, tissue was harvested from the outer molecular layer and the granule cell layer of the denervated dentate gyrus. Expression levels of candidate genes were assessed using Affymetrix GeneChip Mouse Gene 1.0 ST arrays and reverse transcription-quantitative PCR, revealing an upregulation of *Adam10* mRNA and *Adam17* mRNA in the denervated outer molecular layer and an upregulation of *Adam10* mRNA and *App* mRNA in the dentate granule cell layer. Immunolabeling for ADAM10 or ADAM17 in combination with markers for astro- and microglia revealed an increased labeling of ADAM10 and ADAM17 in the denervated outer molecular layer that was associated with reactive astrocytes but not with microglia. Collectively, these data show that denervation affects the expression level of APP and its two most important α-secretases. This suggests that APP-processing could be shifted towards the non-amyloidogenic pathway in denervated areas of the brain and, thus, towards the formation of neuroprotective APP cleavage products, such as APPsα.

## Introduction

Amyloid precursor protein (APP) is centrally involved in the pathogenesis of Alzheimer’s disease (e.g., [1], [2]). It is a transmembrane protein that is proteolytically processed along two alternative pathways (e.g., [3]–[5]): The amyloidogenic pathway results in the production of amyloid-ß (Aß) and requires cleavage of APP via ß- and γ-secretases. This pathway leads to the formation of amyloid fibrils and plaques. In contrast, the non-amyloidogenic pathway results in the production of soluble amyloid precursor protein-α (APPsα) and requires cleavage of APP via α- and γ-secretases. This pathway is believed to play a role in neuronal plasticity [5], [6], neuroprotection [7]–[11] and brain trauma [12]–[15].

It has been suggested that a delicate balance exists between the two pathways, which could depend on the activity of the APP processing secretases (e.g., [16]). Increased ß-secretase activity could lead to an increased production of neurotoxic Aß, while increased α-secretase activity could result in an increased production of neuroprotective APPsα [16]. How brain trauma could affect this delicate balance is controversially discussed in the literature [17]. It has been shown that brain injury can enhance APP expression (e.g., [18]) and that processing of APP along the amyloidogenic pathway can result in an increased Aß deposition in brain [19]. However, a series of recent reports have shown that lack of APP results in larger lesions and poorer behavioral outcomes following focal traumatic brain injury [13]–[15], consistent with early studies demonstrating enhanced acute mortality in kainate injected APP-deficient mice [20]. This effect appeared to depend on the formation of neuroprotective APPsα, since exogenously applied APPsα could rescue the deficits of APP-deficiency [13] and reduced neuronal injury following trauma [21].

In the present study, we have used the entorhinal denervation model [22]–[24], a classical model of structural reorganization after brain trauma, to better understand the role of APP and APP processing in denervated areas of the brain following injury. We hypothesized that APP or its processing enzymes could be regulated in the dentate gyrus following denervation. In the latter case, this could lead to a shift in the balance of APP processing. Accordingly, we not only studied changes in *App* expression but also changes in the expression of the major α- and β-secretases (*Adam10, Adam17; Bace1*) [5], [25]–[30]. APP-gene family members APP-like protein 1 (*Aplp1*) and APP-like protein 2 (*Aplp2*) were also investigated, since APLP-like proteins are known to compensate in part for APP functions [1], [31], [32].

## Materials and Methods

### Ethics Statement

Animal care and experimental procedures were performed in agreement with the German law on the use of laboratory animals (animal welfare act; TierSchG) and approved by the Regierungspräsidium Darmstadt (Permit Number: RP Darmstadt/V54-19c20/15-F6/01).

### Animals

∼ 12 weeks old male C57Bl6/J mice (Janvier, France) were allowed to survive 1, 3, 7, 14 or 28 days after entorhinal denervation. Experiments were designed to analyze control and lesioned animals of the same age (∼16 weeks). All efforts were made to minimize suffering of experimental animals.

### Entorhinal Denervation and Control of Lesion Quality

Unilateral transection of the left perforant path was performed using a wire knife (David Kopf Instruments, USA) as described previously [33]. All surgery was done under deep anesthesia (midazolame (5 mg/kg body weight [bw]), fentanyl (0.05 mg/kg [bw]) and medetomidine (0.5 mg/kg [bw]). Local anesthesia (Mepivacain, 0.1 ml of 1% solution) was applied subcutaneously directly above the skull. The anesthesia was antagonized at the end of surgery by intraperitoneal injection of flumazenil (0.5 mg/kg [bw]), naloxone (0.12 mg/kg [bw]) and atipamezole (2.5 mg/kg [bw]). Placement of the wire knife cut was verified on horizontal sections (25 µm) containing the lesion site and parts of the temporal dentate gyrus. In addition, entorhinal denervation was verified on frontal sections (25 µm) of the hippocampus: Fluoro-Jade C [34], [35] was employed at early time points post lesion to monitor the appearance of degeneration products. Immunohistochemistry for the astrocytic marker glial fibrillary acidic protein (GFAP) [33] and the microglial marker anti-ionized calcium-binding adapter molecule 1 (IBA1) [36] were used to visualize the denervation-induced glial reaction at late time points.

### Laser Microdissection

Mice were killed by an overdose of isoflurane (Abbott, Germany). Brains were rapidly removed from the cranium, embedded in tissue freezing medium and immediately flash-frozen in −70°C isopentane cooled by dry ice. Brains were stored until further processing at −80°C. For laser microdissection, 16 µm thick sections of the septal hippocampus were cut with a cryostat and mounted on polyethylene naphthalene (PEN) membrane slides (Leica Microsystems, Germany). Sections were dried shortly at room temperature, fixed in −20°C cold 75% and 100% ethanol and were then stored at −80°C until further processing. Sections for laser microdissection were stained quickly with 1% cresyl violet staining solution and then dehydrated briefly in 75% and 100% ethanol. Using a Leica LMD6500 system (Leica Microsystems), defined tissue portions of the granule cell layer and the outer molecular layer of the dentate gyrus were collected separately from the same brain sections in lysis buffer (RNeasy Plus Micro Kit; Qiagen, Germany) and transferred to –80°C until further processing.

### RNA Isolation and Verification of RNA Integrity

Total RNA was isolated using the RNeasy Plus Micro Kit (Qiagen) according to the manufacturer’s recommendations. RNA integrity was assessed using the Agilent 2100 Bioanalyzer system and Agilent RNA 6000 Pico Kit (Agilent Technologies, Germany).

### Microarray Analysis

Total RNA from three different animals per time point (control, 1, 3, 7, 14, 28 days post lesion) was amplified using the Ovation Pico WTA System (NuGEN, Netherlands). The Encore Biotin Module Kit (NuGEN) was used for fragmentation and labeling of cDNA for further analysis. Samples were hybridized using Affymetrix GeneChip Mouse Gene 1.0 ST arrays (Affymetrix, USA). All steps were performed in the Microarray Facility Tübingen (MFT Services, Germany). Gene expression data were analyzed using Affymetrix Expression Console software (Affymetrix) and Partek Genomics Suite 6.5 software (Partek, USA). Data were normalized and filtered for transcripts, which were differentially expressed between lesioned animals and control animals. Significance (p≤0.05) was calculated using an analysis of variance (ANOVA). Only transcripts with a change in expression level of at least 2-fold were considered.

### Reverse Transcription-quantitative Polymerase Chain Reaction (RT-qPCR)

Total RNA from four to six different animals (control, 7 days post lesion) was reverse transcribed using High Capacity cDNA Reverse Transcription Reagents Kit (Applied Biosystems, USA) following the manufacturer’s recommendations. cDNA was preamplified before quantitative polymerase chain reaction (qPCR) using TaqMan PreAmp Master Mix (Applied Biosystems). qPCR conditions were carried out using TaqMan Gene Expression Master Mix (Applied Biosystems). Amplification was performed using the StepOnePlus Real-Time PCR System (Applied Biosystems). qPCR products were checked on Agilent DNA 1000 Chips (Agilent Technologies) with the Agilent 2100 Bioanalyzer system to verify product specificity and amplicon size of TaqMan assays (Table 1). No signals were detected in no-template controls. Primer efficiencies and quantification cycle (Cq) values were calculated using LinRegPCR Software (version 12.7) on amplification data exported from the StepOnePlus Software (version 2.2.2). For normalization an index of three reference genes (*Gapdh*, *Pgk1* and *Sdha*) was used. qPCR data were tested for statistical significance (p≤0.05) using the two-tailed t-test.

**Table 1:** TaqMan assays used in the present study.

Gene symbol	Official full name (NCBI)	TaqMan (ABI)	Size (bp)
***Adam10***	a disintegrin and metallopeptidase domain 10	Mm00545742	109
***Adam17***	a disintegrin and metallopeptidase domain 17	Mm00456428	91
***Aplp1***	amyloid beta (A4) precursor-like protein 1	Mm00545893	82
***Aplp2***	amyloid beta (A4) precursor-like protein 2	Mm00507814	67
***App***	amyloid beta (A4) precursor protein	Mm01344172	111
***Bace1***	beta-site APP cleaving enzyme 1	Mm00478664	124
***Gapdh***	glyceraldehyde-3-phosphate dehydrogenase	Mm99999915	107
***Pgk1***	phosphoglycerate kinase 1	Mm00435617	137
***Sdha***	succinate dehydrogenase complex, subunit A	Mm01352366	82

NCBI, National Center for Biotechnology Information; ABI, Applied Biosystems/life technologies.

### Immunofluorescence

Mice were deeply anesthetized with an overdose of pentobarbital (300 mg/kg body weight) and transcardially perfused with 0.9% NaCl followed by 4% paraformaldehyde (PFA) in PBS (pH 7.4). Brains were removed, post-fixed for 4 to 24 h in 4% PFA and sectioned in the coronal plane (35–40 µm) on a cryostat (CM3050 S, Leica) or using a vibratome (VT1000 S, Leica). Free-floating sections containing the dorsal part of the hippocampus were incubated in a blocking buffer containing 0.3% Triton X-100 and 5% bovine serum albumin (BSA) in 0.05 M tris-buffered saline (TBS) for 1 h at room temperature followed by incubation in the primary antibody (diluted in 0.1% Triton X-100 and 1% BSA in 0.05 M TBS) for 2–3 days at 4°C. The following primary antibodies were used for this study at the specified dilutions: anti-GFAP (1∶500, Dako, Germany), anti-IBA1 (1∶500, WAKO, USA), anti-AIF1/IBA1 (1∶100, Abcam, UK), anti-ADAM10 (1∶250, Abcam) and anti-ADAM17/TACE (1∶200, Santa Cruz, USA). After several washes, sections were incubated with the appropriate secondary Alexa-conjugated antibodies (1∶2000, Invitrogen/Molecular Probes, USA) for 3–4 h at room temperature and finally mounted in Fluorescent Mounting Medium (Dako). In control experiments, the primary antibody was omitted and no specific immunofluorescence signal was observed. Nuclei were counterstained with Hoechst 33342 (1∶50000, Sigma) and/or DRAQ5 (1∶10000, eBioscience).

The numbers of GFAP-positive cells expressing ADAM10 or ADAM17 in the outer molecular layer of the dentate gyrus 7 days after entorhinal denervation were estimated using confocal laser scanning microscopy. Dorsal parts of the hippocampus containing the contra- and ipsilateral dentate gyrus (3–4 regions of interest (ROIs) per brain section, 3 mice per group) were used for quantification. Tissue sections from brain levels corresponding to −1.58 mm to −1.94 mm from bregma according to [37] were used. Optical sections that demonstrated ideal penetration of both antibodies and the nuclear counterstain were captured using a Digital Eclipse C1 Plus Modular Confocal System (Nikon Instruments). The same settings for gain, offset, and pinhole aperture were used for each section. For cell counting, the Nikon EZ-C1 3.80 software in combination with Fiji image processing package based on ImageJ software (version 1.47) equipped with the *cell counter* plugin was used. GFAP-positive cell bodies containing a distinct nucleus were selected and counted by an observer blind to the experimental condition. A total of approximately 400 GFAP-positive cells per group were counted to estimate the proportion of GFAP-positive astrocytes that were ADAM10- or ADAM17-positive following entorhinal denervation. Data were tested for statistical significance using the two-tailed t-test (p≤0.05; mean ± SEM).

### Digital Illustrations

Figures were prepared digitally using commercially available graphics software (Photoshop CS6, Adobe Inc.). Fluorescent images were acquired using a digital camera (DS-5Mc, Nikon, Germany) or confocal laser scanning microscopy (Eclipse C1 Plus, Nikon). Single fluorescent images of the same section were digitally superimposed. The contrast, brightness and sharpness of images were adjusted as needed for each section. No additional image alteration was performed.

## Results

### Lesion Control

**Figure 1:**
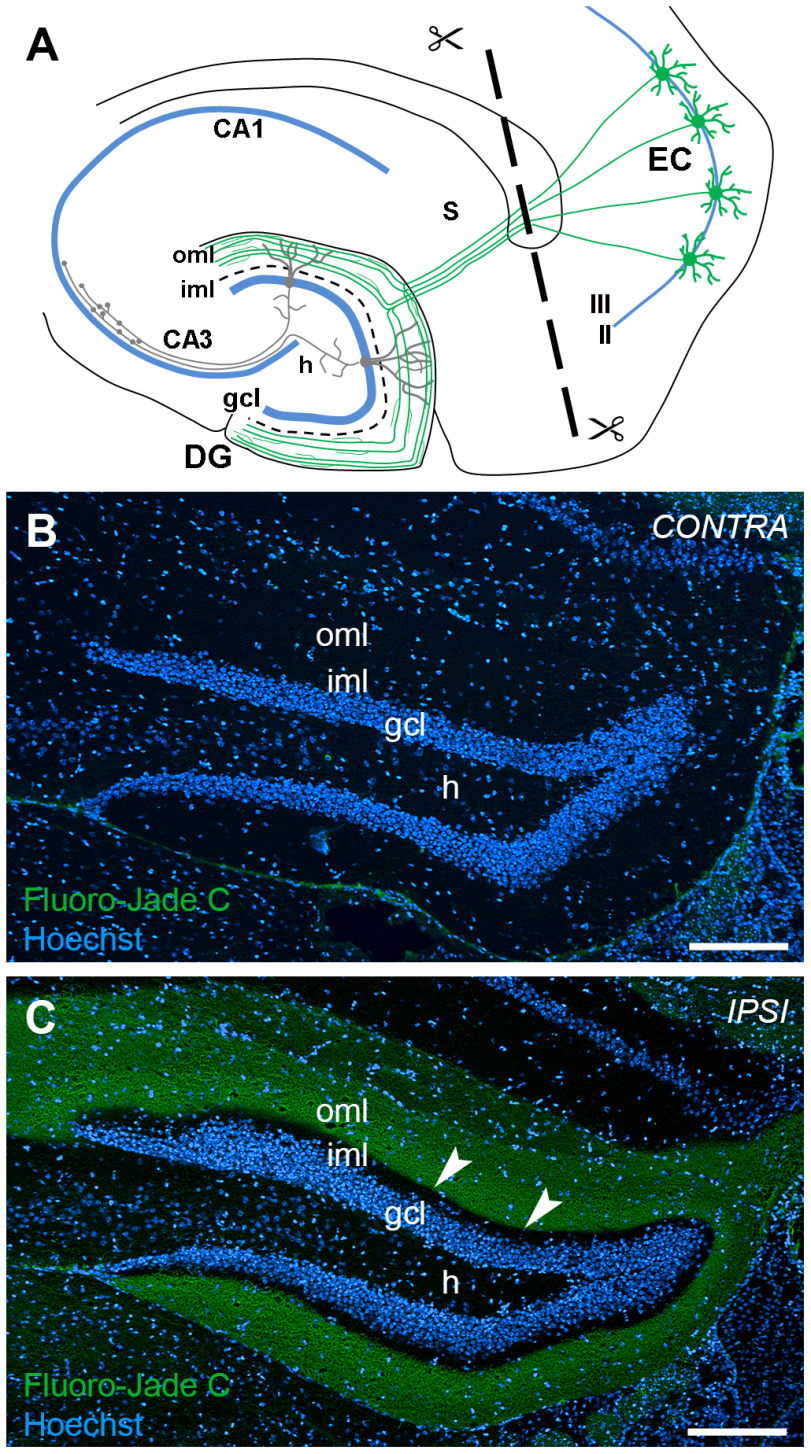
Entorhino-hippocampal denervation model. (A) Schematic of a horizontal brain section illustrating the entorhino-hippocampal denervation model. The perforant path (green) originates from stellate neurons in the entorhinal cortex (EC) and terminates on distal dendritic segments of granule cells (gray) in the outer molecular layer (oml) of the dentate gyrus (DG). The transection site of the perforant path is indicated by a dashed line. (B, C) Layer-specific denervation of the dentate oml following unilateral transection of the perforant path. Fluoro-Jade C staining of the hippocampus contralateral (B) and ipsilateral (C) to the lesion side reveals degenerating axons in denervated areas of the dentate molecular layer at 7 days post lesion. Arrowheads point to the border between the denervated oml and non-denervated inner molecular layer (iml). Nuclei were counterstained with Hoechst 33342. gcl: granule cell layer; h: hilar region. Scale bar: 200 µm.

**Figure 2:**
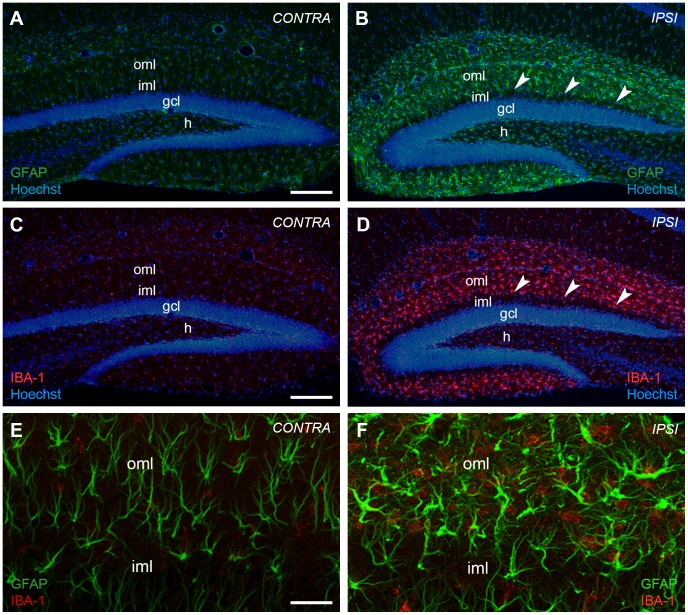
Entorhinal denervation leads to a glial cell reaction in the outer molecular layer of the mouse dentate gyrus. Unilateral transection of the perforant path leads to an activation of glial cells, i.e. astrocytes and microglia, in the denervated outer molecular layer (oml) of the mouse dentate gyrus. The enhanced reactivity of glial cells is shown at 7 days post lesion with immunostainings against glial fibrillary acid protein (GFAP) (B) and ionized calcium-binding adapter molecule 1 (IBA1) (D), marker molecules for reactive astrocytes and activated microglia, respectively. The contralateral hippocampus is shown as control (GFAP (A); IBA1 (C)). Nuclei were counterstained with Hoechst 33342. Arrowheads point to the border between the denervated oml and non-denervated inner molecular layer (iml). (E) and (F) Higher magnification analysis by confocal imaging of tissue sections double-stained for GFAP and IBA1 demonstrates the activation of both glial cell types in the denervated oml. The two cell populations are clearly distinct. gcl: granule cell layer; h: hilar region. Scale bars: (A–D): 200 µm, (E, F): 50 µm.

Wire knife lesions are performed stereotaxically and a considerable degree of variability is to be expected [24]. To ensure that our wire knife lesions selectively transected the perforant path, we took care that lesions were neither too extensive, i.e. injured the hippocampus, nor too small, i.e. incomplete. Only animals in which the hippocampus was undamaged were included in the analysis. The completeness of the lesion was further verified at early time points post lesion using Fluoro-Jade C [34] staining to visualize fiber degeneration (Fig. 1) or - at late time points post lesion - immunohistochemistry for the glial markers GFAP (Fig. 2A, B) and IBA1 (Fig. 2C, D). As expected, these two markers did not colocalize (Fig. 2E, F). On the basis of these criteria we are confident that the entorhinal denervation of the outer molecular layer was complete at the different time points examined.

### Laser Microdissection of Dentate Subregions

**Figure 3:**
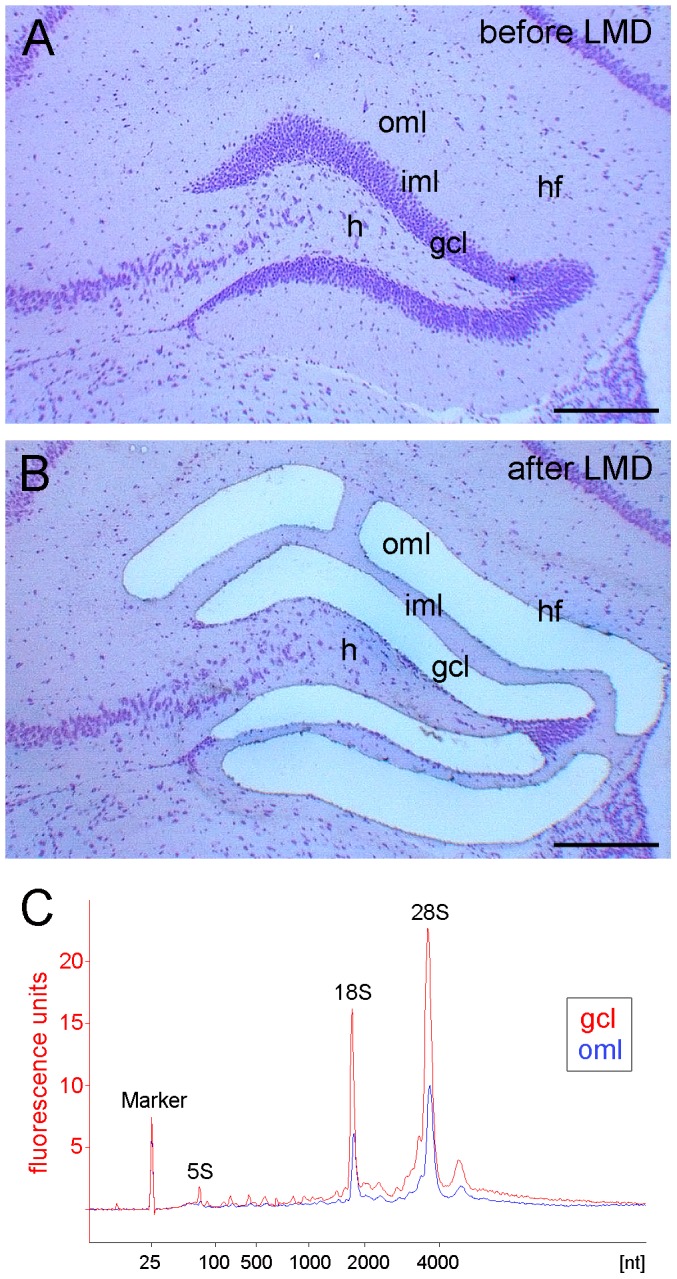
Laser microdissection of dentate subregions. Hippocampal section (coronal plane, dorsal part of the hippocampus, cresyl violet staining) before (A) and after (B) laser microdissection (LMD) of the granule cell layer (gcl) and the outer molecular layer (oml). (C) RNA integrity analysis of total RNA isolated from the dissected granule cell layer (gcl, red) and from the outer molecular layer (oml, blue) demonstrating highly intact RNA (RIN-values: 8.15-8.3; Agilent 2100 Bioanalyzer). hf: hippocampal fissure; iml: inner molecular layer; h: hilar region. Scale bar: 200 µm.

Laser microdissection was used to isolate the outer molecular layer and the granule cell layer of the dentate gyrus (Fig. 3). This approach uses a rapid histological staining and an ultraviolet laser to dissect the tissue in a contact-free environment, thereby ensuring the isolation of high RNA quality for further processing [38]. Moreover, this approach allowed us to dissect the molecular and the granule cell layers from the same control or lesioned animal, thus minimizing biological and technical variability in this study (Fig. 3A, B). RNA integrity analysis of total RNA isolated from the dissected granule cell layer and from the outer molecular layer revealed highly intact RNA with RIN-values >8 (Fig. 3C) as determined by the Agilent 2100 Bioanalyzer system [39].

### Microarray and RT-qPCR Analysis of Dentate Subregions

Microarray analysis using Affymetrix GeneChip Mouse Gene 1.0 ST arrays was employed to study changes in the mRNA expression of the α-secretases *Adam10* and *Adam17*, the ß-secretase *Bace1*, and the *App*-gene family (*App, Aplp1, Aplp2*) after entorhinal denervation. Tissue from three different animals per time point (control, 1, 3, 7, 14, 28 days post lesion) were used for microarray analysis. Only *Adam17* mRNA was found to be differentially expressed with a maximal upregulation in the outer molecular layer on day 7 after entorhinal denervation (∼2.2 fold) (Table 2).

**Table 2:** Microarray analysis of candidate genes in the dentate outer molecular layer and granule cell layer after entorhinal denervation.

Gene	Probeset ID	outer molecular layer (oml)					granule cell layer (gcl)				
		1 dpl	3 dpl	7 dpl	14 dpl	28 dpl	1 dpl	3 dpl	7 dpl	14 dpl	28 dpl
		FC	FC	FC	FC	FC	FC	FC	FC	FC	FC
		p-value	p-value	p-value	p-value	p-value	p-value	p-value	p-value	p-value	p-value
***Adam10***	10586844	1,06	1,26	1,12	1,00	−1,09	1,31	1,04	1,01	1,32	1,19
		0,713	0,194	0,560	0,982	0,643	0,112	0,852	0,977	0,188	0,405
***Adam7***	10399605	1,67	2,03	2,21	1,50	1,51	1,16	−1,06	1,37	1,37	1,36
		0,044	0,010	0,009	0,096	0,127	0,723	0,913	0,570	0,551	0,563
***Aplp1***	10561927	1,22	1,01	1,11	1,04	1,21	1,12	1,09	1,08	1,05	1,03
		0,029	0,879	0,277	0,590	0,051	0,607	0,778	0,791	0,875	0,914
***Aplp2***	10592023	1,04	−1,04	−1,10	1,23	−1,01	−1,10	1,07	−1,01	1,04	1,10
		0,623	0,598	0,265	0,018	0,892	0,684	0,829	0,980	0,901	0,747
***App***	10440491	−1,01	−1,05	−1,16	1,04	−1,03	1,01	1,06	−1,05	1,17	1,12
		0,935	0,413	0,062	0,576	0,717	0,947	0,759	0,786	0,390	0,551
***Bace1***	10584941	1,20	1,26	−1,13	1,11	1,24	2,09	2,04	−1,09	1,48	1,79

dpl, days post lesion; FC, fold change.

**Figure 4:**
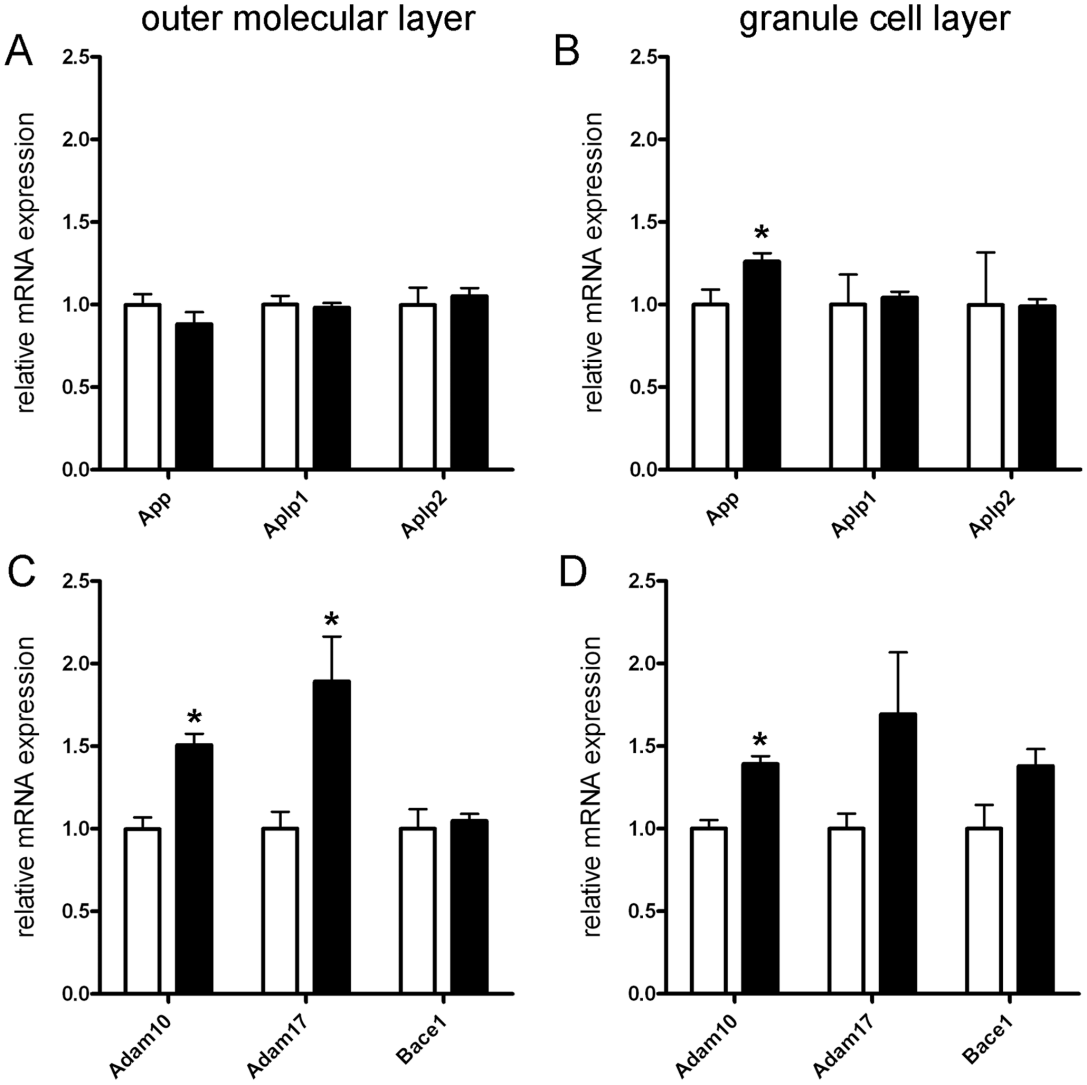
Upregulation of *App*, *Adam10* and *Adam17* mRNAs in the dentate gyrus at 7 days after entorhinal denervation. Expression levels of *App, Aplp1* and *Aplp2* mRNAs were determined by reverse transcription-quantitative polymerase chain reaction (RT-qPCR) in the denervated outer molecular layer (A) and in the granule cell layer (B). Only *App* mRNA was found to be upregulated in the granule cell layer. Similarly, mRNA expression levels of *Adam10*, *Adam17* and *Bace1* were determined by RT-qPCR in the denervated outer molecular layer (C) and in the granule cell layer (D). Significant upregulation of *Adam10* and *Adam17* mRNAs were seen in the denervated outer molecular layer (C). In addition, *Adam10* mRNA was found to be increased in the granule cell layer (D). (n = 4–6 animals each; t-test (two-tailed), * = p≤0.05).

Next, RT-qPCR analysis was performed at 7 days post lesion for all genes studied to validate lesion-associated changes in gene expression obtained by microarray analysis (Fig. 4). In the outer molecular layer, the upregulation of *Adam17* mRNA (∼1.9-fold) could be confirmed at 7 days post lesion (Fig. 4C). In addition, a significant upregulation of *Adam10* mRNA was seen (∼1.5-fold; Fig. 4C). No significant changes in gene expression were revealed for *App*, *Aplp1, Aplp2* and *Bace1* mRNA in the denervated outer molecular layer (Fig. 4A, C). In the granule cell layer a slightly different mRNA expression pattern was seen. Although we also observed an upregulation of *Adam10* mRNA (∼1.4-fold) (Fig. 4D) only a trend for the upregulation of *Adam17* mRNA was seen. Similarly, a trend for the upregulation of *Bace1* mRNA was found, which did not reach the level of significance (Fig. 4D). In contrast to *App* mRNA levels in the outer molecular layer, *App* mRNA was slightly but significantly upregulated in the granule cell layer (∼1.3-fold) (Fig. 4B).

### Reactive Astrocytes Upregulate ADAM10 in the Outer Molecular Layer of the Mouse Dentate Gyrus After Entorhinal Denervation

**Figure 5:**
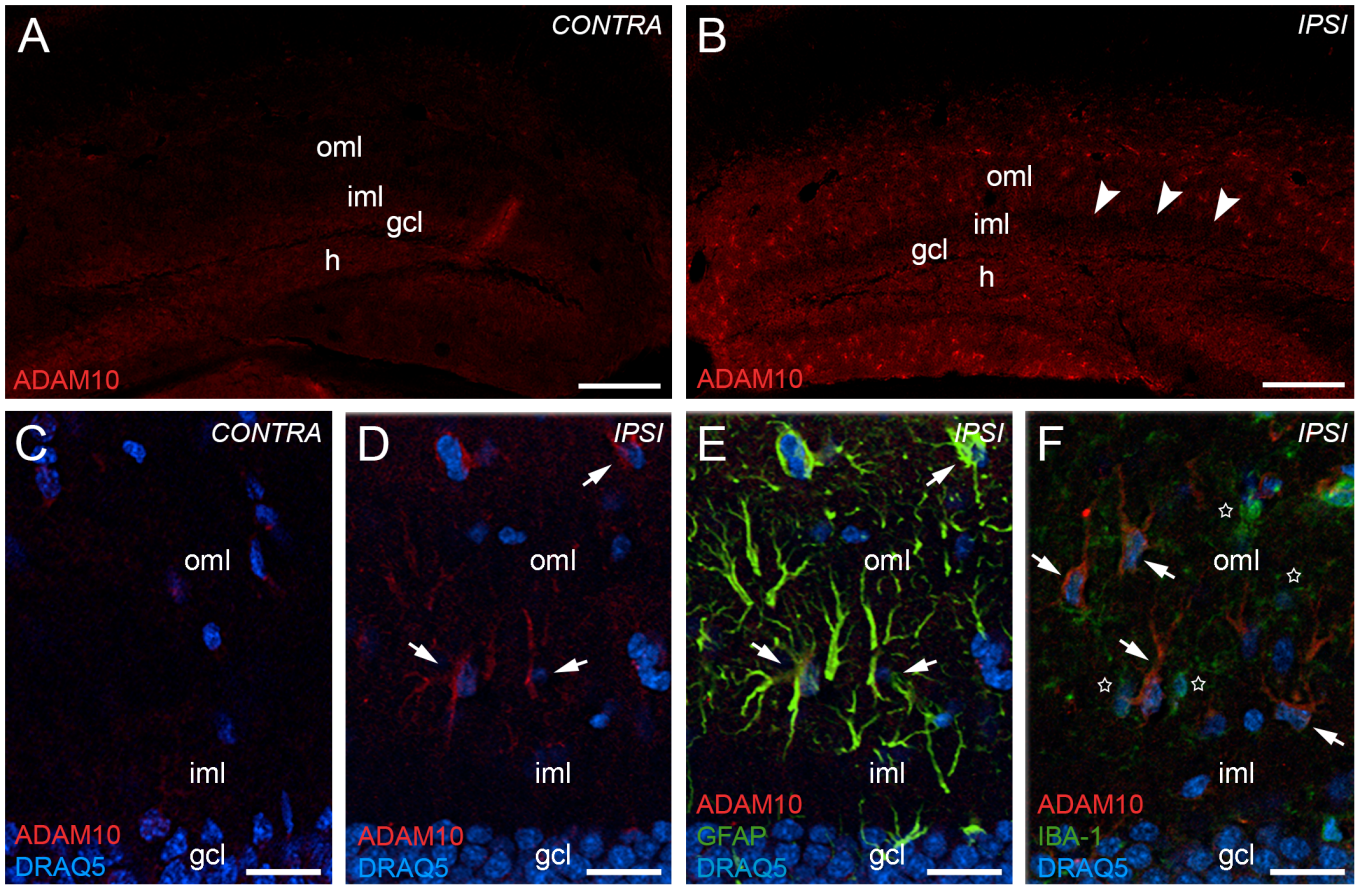
Reactive astrocytes upregulate ADAM10 in the dentate gyrus 7 days after entorhinal denervation. ADAM10 immunofluorescence of the hippocampus contralateral (A, C) and ipsilateral (B, D) to the lesion side revealed an upregulation of ADAM10 in the denervated outer molecular layer (oml) of the dentate gyrus. Arrowheads (in B) point to the border between the denervated oml and the non-denervated inner molecular layer (iml). Confocal analysis of sections double-stained for ADAM10 (E, F; red), the astrocytic marker GFAP (E; in yellow/green) and the microglia marker IBA1 (F; green) revealed that increased ADAM10 immunofluorescence (D–F, arrows) is associated with reactive astrocytes (E, arrows), but not with activated microglia (F, stars) following entorhinal denervation. Nuclei were counterstained with DRAQ5. h: hilar region. Scale bars: (A) 200 µm; (C) 25 µm.

Since our RT-qPCR data indicated an upregulation of *Adam10* mRNA in the denervated dentate gyrus, we wondered whether ADAM10 was also changed at the protein level. Accordingly, we performed immunohistochemistry for ADAM10 (Fig. 5). In the dentate gyrus contralateral to the lesion, which served as a control, only a very weak and diffuse immunostaining was detected in the molecular layer and in the granule cell layer of the dentate gyrus (Fig. 5A, C). In contrast, in the dentate gyrus ipsilateral to the lesion (7 days post lesion) the outer molecular layer was strongly immunoreactive (Fig. 5B, D).

The morphology and pattern of ADAM10-positive cells in the outer molecular layer of lesioned animals closely resembled that reported for reactive astrocytes in the mouse dentate gyrus after denervation [33] (see also Fig. 2F). Therefore, double-immunolabeling for ADAM10 and for the astrocytic marker GFAP was performed. Confocal single section analysis revealed that essentially all ADAM10-positive cells in the outer molecular layer were also GFAP-positive and, thus, could reliably be identified as reactive astrocytes (Fig. 5E). In contrast, double-immunolabeling for ADAM10 and for the microglial marker IBA1 did not reveal colocalization of the two markers (Fig. 5F). Thus, we could exclude that activated microglia contribute to the increased ADAM10-immunoreactivity in the outer molecular layer after entorhinal denervation.

To estimate the percentage of reactive astrocytes expressing ADAM10 immunofluorescence in the outer molecular layer at 7 days post lesion, we quantified the number of GFAP-positive and ADAM10-positive (GFAP+/ADAM10+) and the number of GFAP-positive and ADAM10-negative (GFAP+/ADAM10−) cells using confocal microscopy. A significant (p≤0.05, two-tailed t-test) increase of GFAP+/ADAM10+ cells was observed in the ipsilateral outer molecular layer (100±0.0%) compared to the contralateral outer molecular layer (11.8±1.6%) of the dentate gyrus following entorhinal denervation.

### Reactive Astrocytes Upregulate ADAM17 in the Outer Molecular Layer of the Mouse Dentate Gyrus After Entorhinal Denervation

**Figure 6:**
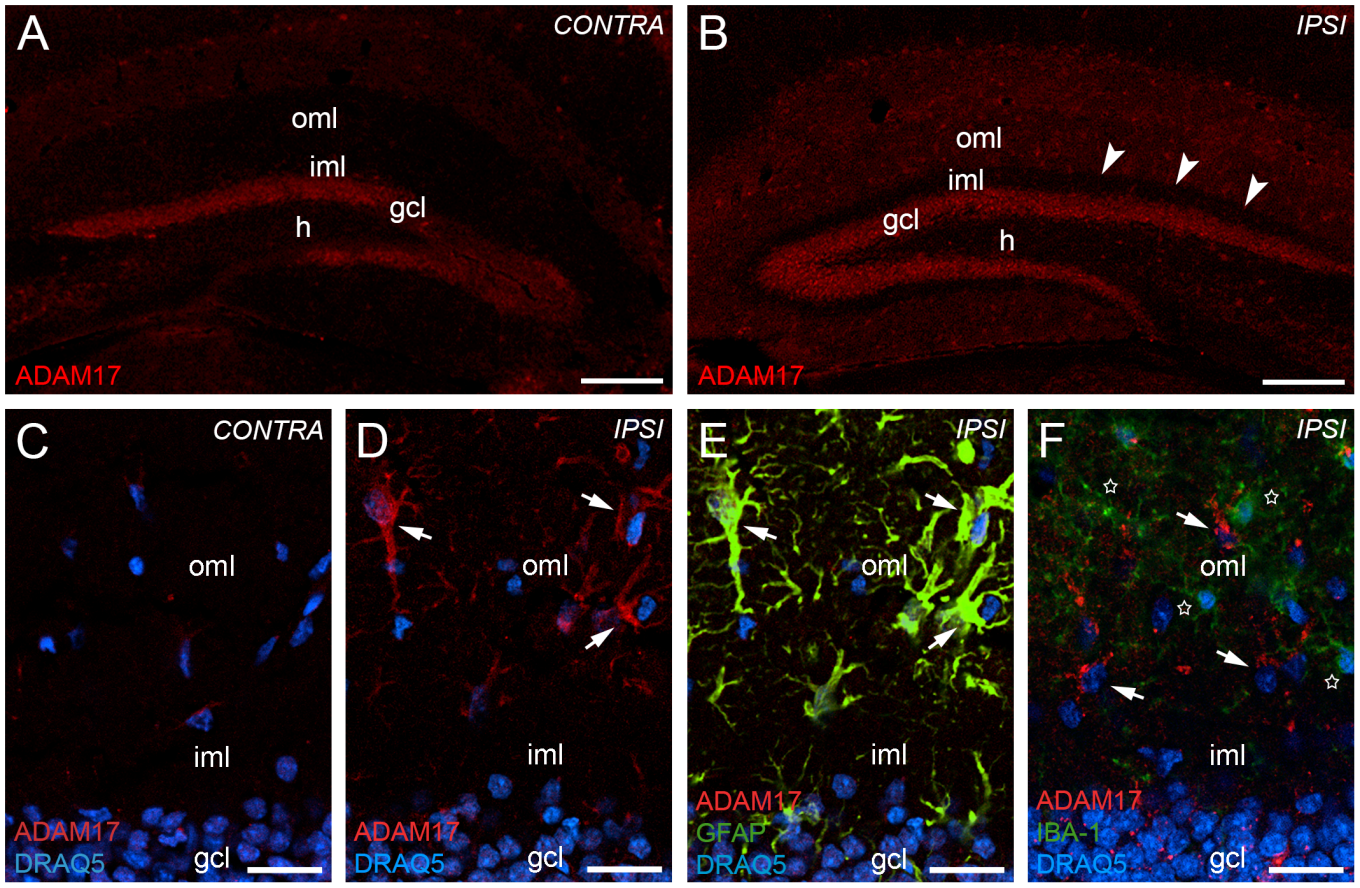
Reactive astrocytes upregulate ADAM17 in the dentate gyrus 7 days after entorhinal denervation. ADAM17 immunolabeling of the hippocampus contralateral (A, C) and ipsilateral (B, D) to the lesion side revealed an upregulation of ADAM10 in the denervated outer molecular layer (oml) of the dentate gyrus. Arrowheads point to the border between the denervated oml and the non-denervated inner molecular layer (iml). Confocal analysis of sections double-stained for ADAM17 (E, F; red), the astrocytic marker GFAP (E; in yellow/green) and the microglia marker IBA1 (F; green) revealed that increased ADAM17 immunofluorescence (D–F, arrows) is associated with reactive astrocytes (E, arrows), but not with activated microglia (F, stars) following entorhinal denervation. Nuclei were counterstained with DRAQ5. h: hilar region. Scale bars: (A) 200 µm; (C) 25 µm.

To study the cellular expression of ADAM17 on the protein level, a similar approach was used. In the dentate gyrus contralateral to the lesion side, immunoreactivity for ADAM17 was seen primarily in the granule cell layer of the dentate gyrus (Fig. 6A). In the molecular layer immunoreactivity for ADAM17 was very weak (Fig. 6A, C). After 7 days post lesion, the outer molecular layer of the dentate gyrus ipsilateral to the lesion side showed a strong increase in immunolabeling for ADAM17. The non-denervated inner molecular layer was ADAM17-poor (Fig. 6B, D).

The staining pattern of ADAM17-positive cells in the dentate outer molecular layer (Fig. 6D) again resembled that of reactive astrocytes after entorhinal denervation and, indeed, using a confocal double-labeling strategy, we could demonstrate that these cells were GFAP-positive (Fig. 6E) and IBA1-negative (Fig. 6F). Thus, ADAM17-positive cells appear to be reactive astrocytes and not activated microglia.

To estimate the percentage of reactive astrocytes expressing ADAM17 immunofluorescence in the outer molecular layer at 7 days post lesion, we quantified the number of GFAP-positive and ADAM17-positive (GFAP+/ADAM17+) and the number of GFAP-positive and ADAM17-negative (GFAP+/ADAM17−) cells using confocal microscopy. A significant (p≤0.05, two-tailed t-test) increase of GFAP+/ADAM17+ cells was observed in the ipsilateral outer molecular layer (98.6+/−1.4%) compared to the contralateral outer molecular layer (9.3+/−2.5%) of the dentate gyrus in dorsal parts of the hippocampus following entorhinal denervation.

## Discussion

In the present study, we analyzed the expression of the *App*-gene family (*App, Aplp1, Aplp2*), *Adam10*, *Adam17* and *Bace1* in the dentate gyrus following entorhinal denervation. Our main findings can be summarized as follows: Entorhinal denervation caused a transient upregulation of *Adam10* mRNA and *Adam17* mRNA in the denervated outer molecular layer of the dentate gyrus and of *Adam10* mRNA and *App* mRNA in the granule cell layer. At the protein level, upregulation of ADAM10 and ADAM17 was seen in reactive astroglia but not in activated microglia at 7 days post lesion. In contrast, *Bace1* mRNA showed no significant change in expression after denervation. Taken together, these data suggest that processing of APP could be shifted towards the non-amyloidogenic pathway in the denervated dentate gyrus.

### ADAM10 and ADAM17 are Expressed by Reactive Astrocytes in the Denervated Outer Molecular Layer

Our data show that the mRNAs for the α-secretases *Adam10* and *Adam17* are upregulated in the denervated outer molecular layer of the dentate gyrus. In this layer, astrocytes and microglia react strongly to denervation and become activated [23], [24], [33], [40]–[45]. Accordingly, we speculated that astroglia or microglia could be the α-secretase expressing cells and performed double-immunofluorescence for ADAM10, ADAM17 and glial markers. These experiments revealed that astrocytes but not microglia are positive for both α-secretases after entorhinal denervation.

In a recent report following entorhinal denervation in rats [46] changes in ADAM10 protein but no changes in *Adam10* mRNA were reported in the deafferented dentate molecular layer at 7 days after unilateral entorhinal cortex lesion. Although species differences may play a role [24], the techniques used in the present study (i.e. laser microdissection of the denervated layer in combination with RT-qPCR; [38]) are very sensitive and allow for the detection of increases in mRNA expression which may be missed otherwise. Thus, our data provide first evidence that entorhinal denervation upregulates *Adam10* and *Adam17* mRNAs in the mouse dentate gyrus.

The observed colocalization of ADAM10 and astrocytes is in line with observations made by Warren and coworkers (2012) in rats. We confirm this finding for the mouse brain and provide additional data for ADAM17, which may also act as α-secretase [5], [27], [47]. Both α-secretases are strongly expressed by reactive astrocytes but apparently not by activated microglia. Reactive astrocytes are located within the layer of fiber degeneration and reactive synaptogenesis, and they have been shown to play a major role in the reorganization of the dentate gyrus following denervation (e.g., [23], [40], [45], [48]–[50]). Their distal processes are elements of the tripartite synapse [51], [52] and, thus, reactive astrocytes are in an ideal position to process the extracellular matrix (e.g., [40], [53]–[55]) or adhesion molecules such as N-cadherin [46] and/or APP [56], [57] at or in the vicinity of synapses.

### *App* mRNA and *Adam10* mRNA are Upregulated in the Granule Cell Layer Following Entorhinal Denervation

APP expression changes have been previously studied in several species using different brain injury models [17], [18], [58], [59]. Collectively, these publications report an increased expression of *App* mRNA in the vicinity of brain injury sites and an accumulation of APP protein. Since brain trauma is regarded as a risk factor for Alzheimer’s disease [60], an upregulation of *App* mRNA and APP protein following brain injury appears to be a plausible link. However, in the light of our current knowledge about APP processing, it has to be kept in mind that increased levels of APP could be processed either along the amyloidogenic or along the non-amyloidogenic pathway, depending on the balance between the APP-processing secretases. As suggested by the work of Corrigan and coworkers (2012), APP upregulation at injury sites could also be neuroprotective, if APP is primarily processed along the non-amyloidogenic pathway.

Compared to the number of reports on APP expression changes at the site of a brain injury, far less data are available on APP changes in denervated brain areas. In a previous study performed in rats [61], changes in hippocampal *App* mRNA expression were reported 7 days after entorhinal cortex lesion using in situ hybridization [61]. In this earlier study, a significant increase in *App* mRNA was found in Ammon’s horn of the hippocampus and a trend towards higher *App* mRNA levels was seen in the dentate gyrus. In our study we detected a small but significant increase in *App* mRNA in the granule cell layer at 7 days after entorhinal denervation using the laser microdissection technique in combination with qPCR, thus confirming the trend seen by Ramirez et al. (2001). Of note, this increase in *App* mRNA expression was accompanied by an increase of *Adam10* mRNA in the same layer. A concomitant increase of APP and α-secretase expression has also been commonly seen in vitro, suggesting that under such conditions APP family members are preferentially cleaved by ADAM10, rather than other ADAM10 substrates (see [62], for review). Taken together, this argues for an altered expression and processing of APP by dentate granule cells following entorhinal denervation.

### APP Processing may be Shifted Towards the Non-amyloidogenic Pathway in the Denervated Dentate Gyrus

Following entorhinal denervation, we observed a moderately increased mRNA expression of *App* and the α-secretases *Adam10* and *Adam17* without a significant increase in mRNA expression levels of the β-secretase *Bace1*. Since *Adam10* mRNA levels translate into protein and result in an increased α-secretase activity [62]–[65], this pattern of mRNA expression argues for a shift in the balance of APP processing in favor of the non-amyloidogenic pathway, i.e. towards an increased secretion of APPsα. The moderate increase in *Adam10* mRNA expression observed in our study is in line with these earlier reports in which it has been shown that a moderate upregulation of *Adam10* mRNA in vivo (∼130%; [65]) results in increased ADAM10 levels (∼130%; [66]) and a relevant increase in α-secretase activity [66]. In contrast, a slightly higher expression (∼170%) of ADAM10 in vivo was detrimental and led to alterations in myelin [67]. Thus, the degree of upregulation of *Adam10* mRNA observed in our study appears to be within the range of transcriptional regulation for this gene.

The balance of APP processing also depends on the activity of the β-secretase BACE1 [2], [3], [68], [69]. Following entorhinal denervation, *Bace1* mRNA levels were not significantly increased, indicating that BACE1 is not regulated at the transcriptional level in this model. However, BACE1 is regulated post-transcriptionally [70] and APPsα has emerged as a potent post-transcriptional regulator of BACE1 activity [71], [72]. In line with this, overexpression of ADAM10 in vivo resulted in an inhibition of Aß production, most likely via increased levels of APPsα ([66].; reviewed in [63]). An increase in *Adam10* mRNA as observed in our study could similarly affect BACE1 activity via increased APPsα levels, thereby shifting the balance of APP processing further towards the non-amyloidogenic pathway.

At present it is very difficult to provide direct proof for the above hypothesis at the protein level. As has been noted by others, it is “nearly impossible to determine endogenous mouse APPsα levels and a potential increase via over-expression of ADAM10” [73]. Although we attempted to measure APPsα levels directly in microdissected material of the hippocampus, we failed to detect an APPsα signal in this tissue (data not shown). More sensitive tests for this biologically highly relevant molecule will have to be developed to successfully address this question.

### ADAM10 and ADAM17 are Enriched in the Zone of Neuronal Reorganization

What may be the functional relevance of an increased expression of ADAM10 and ADAM17 in the outer molecular layer following entorhinal denervation? As has been pointed out in several recent reviews, ADAM10 and ADAM17 have multiple substrates in addition to APP [26]–[28] and many of these molecules, i.e. cell adhesion molecules, extracellular matrix molecules, and cytokines, are found in the denervated outer molecular layer before and after denervation [23], [40]. Thus, an increased expression of ADAM10 and ADAM17 is likely to have pleiotropic effects on the reorganization process, as has been previously suggested for ADAM10 [46], [55].

As far as ADAM10 and ADAM17-dependent processing of APP is concerned, however, an increased upregulation and processing of APP along the non-amyloidogenic pathway could protect granule cells from denervation-induced stress and could beneficially affect dendritic reorganization processes following entorhinal denervation. Entorhinal denervation in mice causes a denervation-induced dendritic atrophy and transient spine loss [35]. These neuronal changes are, however, limited in their extent [35]. Increased levels of neuroprotective APPsα could help to protect denervated neurons from progressive and more extensive damage. This concept would be in agreement with the well-established neuroprotective function of APPsα [8], [9] and the recent in vivo data of Corrigan and co-workers [13]–[15], who observed more extensive neuronal damage following brain injury in mice lacking APP.
